# Treatment Maneuvers in Cupulolithiasis of the Posterior Canal Benign Paroxysmal Positional Vertigo: A Randomized Clinical Trial

**DOI:** 10.1001/jamanetworkopen.2025.0972

**Published:** 2025-03-19

**Authors:** Eun Hye Oh, Jae-Hwan Choi, Hyun Sung Kim, Seo Young Choi, Hyun Ah Kim, Hyung Lee, In Soo Moon, Ji-Yun Park, Byeol-A Yoon, Sang Ho Kim, Jeong-Yeon Kim, Hyo Jung Kim, Kwang-Dong Choi

**Affiliations:** 1 Department of Neurology, Pusan National University School of Medicine, Research Institute for Convergence of Biomedical Science and Technology, Pusan National University Yangsan Hospital, Yangsan, South Korea; 2 Department of Neurology, Gyeongsang National University Changwon Hospital, Changwon, South Korea; 3 Department of Neurology, Pusan National University Hospital, Pusan National University School of Medicine and Biomedical Research Institute, Busan, South Korea; 4 Department of Neurology, Keimyung University Dongsan Hospital, Daegu, South Korea; 5 Department of Neurology, Daedong Hospital, Busan, South Korea; 6 Department of Neurology, University of Ulsan College of Medicine, Ulsan University Hospital, Seoul, South Korea; 7 Department of Neurology, Dong-A University Hospital, Busan, South Korea; 8 Kim’s Neurology Clinic, Busan, South Korea; 9 Research Administration Team, Seoul National University Bundang Hospital, Seongnam, South Korea

## Abstract

**Importance:**

Head-shaking and mastoid oscillation maneuvers effectively treat cupulolithiasis of horizontal canal benign paroxysmal positional vertigo. However, to date there are no validated treatments for cupulolithiasis of posterior canal benign paroxysmal positional vertigo (PC-BPPV-cu).

**Objective:**

To determine the immediate and short-term therapeutic efficacy of head-shaking and mastoid oscillation maneuvers in PC-BPPV-cu.

**Design, Setting, and Participants:**

This double-blind randomized clinical trial was conducted at 6 referral-based university hospitals in South Korea between November 1, 2019, and April 30, 2023. The inclusion criteria consisted of (1) repetitive episodes of positional vertigo or dizziness; (2) positional nystagmus beating torsionally with the upper pole of the eye to the lower ear and vertically upward (to the forehead) and lasting longer than 1 minute, which was evoked by Dix-Hallpike or half Dix-Hallpike maneuver; and (3) absence of accompanying neurologic symptoms or signs suggesting central nervous system disorders. Patients were excluded who declined to participate or had cervical spine problems, multicanal BPPV, or cognitive dysfunction.

**Interventions:**

Patients were randomly assigned to the head-shaking, mastoid oscillation, or sham maneuver groups. After a maximum of 2 trials of each maneuver, therapeutic response was assessed within 30 minutes and the following day.

**Main Outcomes and Measures:**

The primary outcome was the short-term resolution rate of positional vertigo and nystagmus the following day. The secondary outcome was the immediate efficacy of 2 trials of each maneuver within 30 minutes.

**Results:**

A total of 159 patients were included in the analysis (108 [67.9%] women; mean [SD] age, 65.4 [10.5] years). Fifty-three participants were randomized to the head-shaking group (mean [SD] age, 64.0 [11.2] years; 33 [62.3%] women), 53 to the mastoid oscillation group (mean [SD], age, 66.5 [11.1] years; 41 [77.4%] women), and 53 to the control group (mean [SD] age, 65.6 [9.2] years; 34 [64.2%] women). A total of 142 patients (89.3%) completed the assessment the following day. In the intention-to-treat analysis, 20 patients in the head-shaking group (37.7%), 14 in the mastoid oscillation group (26.4%), and 7 in the control group (13.2%) showed the resolution of vertigo and nystagmus the following day (χ^2^ = 8.40; odds ratio, 2.86; 95% CI, 1.32-6.18; *P* = .004; α = .0167). For the secondary outcome (the immediate effectiveness of 2 trials of each maneuver compared with sham within 30 minutes), no significant difference was detected (6 of 53 [11.3%] vs 4 of 53 [7.5%] vs 2 of 53 [3.8%]; *P* = .34; α = .05).

**Conclusions and Relevance:**

In this randomized clinical trial, the head-shaking maneuver was effective in the treatment of PC-BPPV-cu.

**Trial Registration:**

CRIS clinical trial registration: KCT0004756

## Key Points

**Question:**

Are the head-shaking and mastoid oscillation maneuvers effective in the treatment of cupulolithiasis of the posterior canal benign paroxysmal positional vertigo (PC-BPPV-cu)?

**Findings:**

In this randomized clinical trial of 159 patients, patients in the head-shaking group showed a greater short-term therapeutic efficacy (38%) than those in the mastoid oscillation (26%) and control (13%) groups.

**Meaning:**

In this trial, the head-shaking maneuver was effective in treating PC-BPPV-cu; this finding suggests that it can be recommended as an initial treatment option.

## Teaser

This randomized clinical trial assesses the effectiveness of head-shaking and mastoid oscillation maneuvers compared with a sham maneuver in patients with cupulolithiasis of posterior canal benign paroxysmal positional vertigo.

## Introduction

Benign paroxysmal positional vertigo (BPPV) is one of the most common causes of vertigo, particularly in older adults. It occurs when calcium carbonate crystals, known as otoconia, become dislodged from their usual position in the utricle and migrate into one of the semicircular canals. BPPV is often classified into 2 main types based on the location of the displaced crystals: canalolithiasis (otoconia free-floating within the semicircular canal) and cupulolithiasis (otoconia attached to the cupula).^1^

The Bárány Society formulated the diagnostic criteria for cupulolithiasis of the posterior canal benign paroxysmal positional vertigo (PC-BPPV-cu) in 2015.^1^ PC-BPPV-cu produces a positional nystagmus, with the upper pole of the eye beating torsionally toward the lower ear and vertically upward, which lasts longer (>1 minute) than experienced in canalolithiasis of PC-BPPV (PC-BPPV-ca).^1,2,3,4,5,6,7,8,9^ Since there are currently no validated methods for treating PC-BPPV-cu, a 2022 clinical guideline did not advocate specific treatment options based on the subtypes of PC-BPPV (canalolithiasis or cupulolithiasis).^10^

The cupula mainly consists of mucopolysaccharide and is adhesive in nature, which facilitates easy attachment of the otoconia, leading to cupulolithiasis.^3^ Since the therapeutic goals of cupulolithiasis maneuvers are to dislodge otolithic debris from the cupula, head-shaking and mastoid oscillation maneuvers could be adopted for treating cupulolithiasis.^11,12,13^ Vibratory oscillation could settle the particles into the utricle, while acceleration and deceleration of the head could release particles adhered to the cupula.^11,12,13,14^ In experimental models of cupulolithiasis in a bullfrog labyrinth, the otoconial mass was detached in all of 10 labyrinth preparations in the vibration group.^13^

Previous randomized clinical trials^11,12,14^ have proven that head-shaking and mastoid oscillation maneuvers effectively treat cupulolithiasis of horizontal canal benign paroxysmal positional vertigo (HC-BPPV-cu) compared with a sham maneuver. However, no data exist regarding the therapeutic efficacy of these maneuvers in PC-BPPV-cu. We conducted this randomized clinical trial in patients with PC-BPPV-cu to investigate the immediate and short-term therapeutic efficacy of head-shaking and mastoid oscillation maneuvers compared with a sham maneuver.

## Methods

### Study Design, Population, and Randomization

We conducted this multicenter randomized double-blind clinical trial to assess the effectiveness of head-shaking and mastoid oscillation maneuvers compared with the sham maneuver in patients with PC-BPPV-cu at 6 medical centers in South Korea. Patients were screened for participation between November 1, 2019, and April 30, 2023. The trial protocol (Supplement 1) was designed by the principal investigator (K.-D. C.) and approved by the institutional review board at each participating center. Participants’ safety and the benefit-risk balance were overseen according to the data safety monitoring plan. All participants provided written informed consent. The trial was conducted in accordance with Good Clinical Practice guidelines^15^ and the principles of the Declaration of Helsinki.^16^ The Consolidated Standards of Reporting Trials (CONSORT) reporting guideline was followed.

Patients were eligible for inclusion if they had a diagnosis of PC-BPPV-cu according to the diagnostic criteria of the Classification Committee of Vestibular Disorders of the Bárány Society in 2015.^1^ The inclusion criteria were (1) repetitive episodes of positional vertigo or dizziness; (2) positional nystagmus beating torsionally with the upper pole of the eye to the lower ear and vertically upward (to the forehead) and lasting longer than 1 minute, which was evoked by the Dix-Hallpike or half Dix-Hallpike maneuver; and (3) absence of accompanying neurologic symptoms or signs suggesting central nervous system disorders. We excluded patients who were younger than 20 years, declined to participate, or had multicanal BPPV, cervical spine problems, or cognitive dysfunction. All patients were Korean.

Patients were randomly assigned to the head-shaking, mastoid oscillation, or control groups using an interactive web response system provided by Research Electronic Data Capture, version 9.5.2 (Vanderbilt University). Randomization was performed centrally to ensure balanced allocation of patients across the participating centers. The random allocation was designed in permuted blocks with a block size of 3. The investigators assessing the outcomes and analyzing the data were independent and blinded to the patients’ information. In addition, patients were not informed of the treatment they were receiving. To maintain blinding, the neurologist was responsible for conducting positional assessments and documenting the results, acting independently of the physician who administered the treatment.

### Procedures

**Figure 1.**
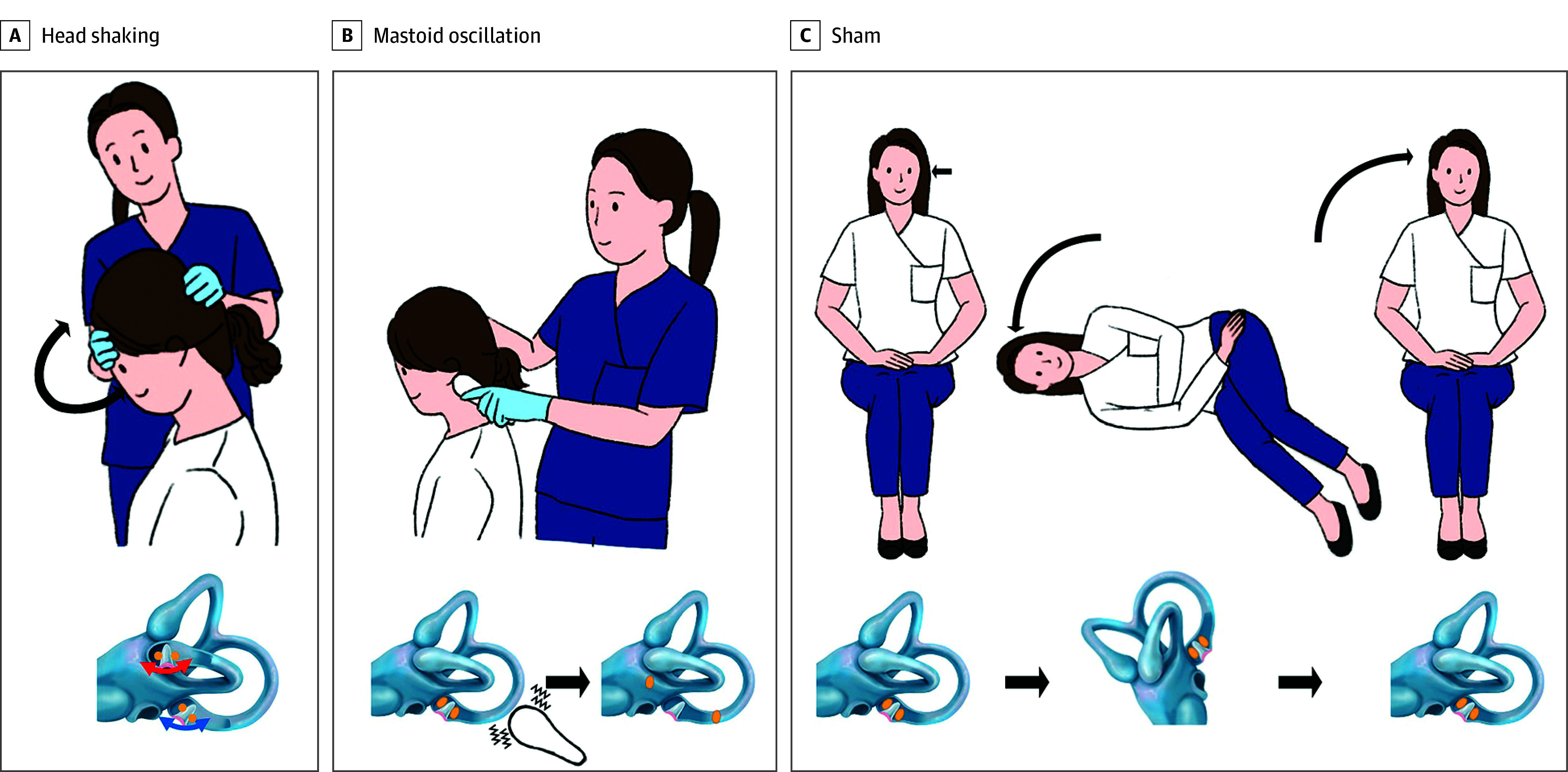
Schematic Drawing of the Study Treatments for Cupulolithiasis of Left Posterior Canal Benign Paroxysmal Positional Vertigo A, In the head-shaking maneuver, the patient is in a sitting position, and after pitching the head forward by 30°, we move patient’s head laterally at approximately 3 Hz for 15 seconds, which detaches otolith from the cupula of the left posterior semicircular canal. The ampulla of the horizontal semicircular canal is almost perpendicular to the ground, while the ampulla of the posterior semicircular canal tilts posteriorly. B, Mastoid oscillation is applied on the mastoid area of the lesion side (left) with a 100-Hz handheld vibrator in the sitting position for 30 seconds. C, For the sham maneuver, the patient is promptly positioned on the unaffected side (right) and then returned to the sitting position after 1 minute. Illustration reproduced with permission of Minkyeong Kim.

A treatment maneuver was performed by a physician according to the assigned treatment or sham groups. For the head-shaking maneuver,^11^ patients were placed in a sitting position. After pitching the head forward by 30°, the head was moved laterally at a sinusoidal rate of approximately 3 Hz for 15 seconds (Figure 1A). For the mastoid oscillation maneuver, the mastoid oscillation was applied to the mastoid area of the lesion side with a 100-Hz handheld vibrator (ID 113; Coms) in a sitting position for 30 seconds (Figure 1B). For the sham maneuver, patients lay on the unaffected side and returned to the sitting position after 1 minute (Figure 1C).

Treatment response was determined by the participating neurologists at each clinic without knowing the maneuver applied to each patient 30 minutes after the initial maneuver. The absence of both vertigo and nystagmus was required to determine a resolution. If the patient had positional vertigo and nystagmus, the previously applied maneuver was repeated. The patients underwent reexamination 30 minutes later and were scheduled for follow-up the next day without additional treatment if they still presented with positional vertigo and nystagmus.

### Outcomes

The primary end point was the short-term resolution rate of positional vertigo and nystagmus the following day. The secondary end point was the immediate efficacy of 2 trials of each maneuver within 30 minutes.

### Statistical Analysis

Based on data from a previously published study,^14,17^ we anticipated a treatment effect size (Cohen h) of approximately 0.65, reflecting the difference in short-term resolution rate of PC-BPPV between the active treatment (70%) and sham treatment (38%) groups. To control for multiple comparisons among the 3 groups, the Bonferroni-adjusted significance level (α = .0167; 2-sided) was required for each treatment arm.

The demographic distribution of study participants by treatment maneuvers was compared using the χ^2^ test and one-way analyses of variance with significance level of *P* < .05. If the results of the χ^2^ test were significant, additional post hoc tests were performed using Bonferroni adjusted α level of .0167 per test (.05/3). Logistic regression analysis was conducted to assess the impact of age and sex on treatment success. Statistical analyses were performed using SPSS, version 27.0 (IBM Corporation).

The primary outcome was analyzed using both intention-to-treat and per-protocol methods. Patients who did not complete the assessment the following day were excluded from the per-protocol analysis. In the intension-to-treat analysis, patients who were lost to follow-up were considered to have treatment failure.

## Results

**Figure 2.**
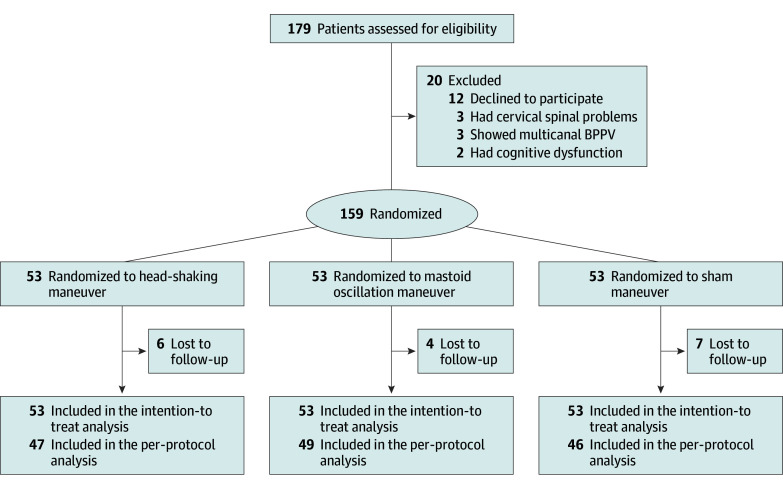
Flowchart for the Enrollment, Randomization, Follow-Up, and Analyses BPPV indicates benign paroxysmal positional vertigo.

In total, 179 patients were assessed for eligibility, of whom 20 (11.2%) were excluded, including those who declined to participate in this study (12 [6.7%]), had cervical spine problems (3 [1.7%]), showed multicanal BPPV (3 [1.7%]), or had cognitive dysfunction (2 [1.1%]). Thus, 159 patients were randomized in the head-shaking (n = 53), mastoid oscillation (n = 53), or sham (n = 53) groups (Figure 2). Patients included 51 men (32.1%) and 108 women (67.9%) with a mean (SD) age of 65.4 (10.5) years. The baseline characteristics were similar among the 3 groups, with a mean (SD) age of 64.0 (11.2) years (33 [62.3%] women) in the head-shaking group, 66.5 (11.1) years (41 [77.4%] women) in the mastoid oscillation group, and 65.6 (9.2) years (34 [64.2%] women) in the sham group (Table 1). A total of 142 patients (89.3%) completed the assessment the following day, while 17 patients (10.7%) were lost to follow-up. There was no significant difference in the proportion of follow-up losses between the treatment and control groups (*P* = .63; α = .05). Specifically, 6 patients in the head-shaking group (11.3%), 4 in the mastoid oscillation group (7.5%), and 7 in the sham group (13.2%) were lost to follow-up or discontinued the intervention due to severe vertigo, anxiety, or vomiting.

**Table 1.** Baseline Characteristics of the Patients

Characteristic	Patient group			
	Head shaking (n = 53)	Mastoid oscillation (n = 53)	Sham (n = 53)	All (N = 159)
Age, mean (SD), y	64.0 (11.2)	66.5 (11.1)	65.6 (9.2)	65.4 (10.5)
Sex, No. (%)				
Men	20 (37.7)	12 (22.6)	19 (35.8)	51 (32.1)
Women	33 (62.3)	41 (77.4)	34 (64.2)	108 (67.9)
Duration of vertigo until evaluation, mean (SD), d	2.8 (3.9)	3.9 (7.4)	1.9 (2.2)	2.9 (5.0)
Affected ear, No. (%)				
Right	27 (50.9)	29 (54.7)	22 (41.5)	78 (49.1)
Left	26 (49.1)	24 (45.3)	31 (58.5)	81 (50.9)
History of BPPV, No. (%)	6 (11.3)	8 (15.1)	9 (17.0)	23 (14.5)

Abbreviation: BPPV, benign paroxysmal positional vertigo.

In the intention-to-treat analysis, 20 patients in the head-shaking group (37.7%), 14 in the mastoid oscillation group (26.4%), and 7 in the control group (13.2%) showed the resolution of vertigo and nystagmus the following day after application of each maneuver (Table 2). The head-shaking group had greater therapeutic efficacy than the sham group (χ^2^ = 8.40; odds ratio [OR], 2.86; 95% CI, 1.32-6.18; *P* = .004; α = .0167). There were no significant differences in the therapeutic efficacy between the head shaking and mastoid oscillation groups (*P* = .21; α = .0167) or between the mastoid oscillation and sham groups (*P* = .09; α = .0167) (Table 3). The groups that received the head-shaking or mastoid oscillation procedures showed higher therapeutic efficacy compared to the sham group (34 [32.1%] vs 7 [13.2%]; *P* = .01; α = .05) (Table 3).

**Table 2.** Resolution Rates of Positional Vertigo and Nystagmus After Each Maneuver

Application time	Patient group, No. (%)			*P* value
	Head shaking (n = 53)	Mastoid oscillation (n = 53)	Sham (n = 53)	
Day 1				
All	6 (11.3)	4 (7.5)	2 (3.8)	.14
First application	4 (7.5)	2 (3.8)	2 (3.8)	NA
Second application	2 (3.8)	2 (3.8)	0	NA
Transition	1 (1.9)	0	0	NA
Day 2				
All	14 (26.4)	10 (18.9)	5 (9.4)	.16
Transition	0	0	2 (3.8)	NA
All	20 (37.7)	14 (26.4)	7 (13.2)	.03
Transition	1 (1.9)	0	2 (3.8)	NA

Abbreviation: NA, not applicable.

**Table 3.** Post Hoc Tests of the Resolution Rates of Positional Vertigo and Nystagmus Between Treatment and Sham Groups

Group comparison	Total resolution rate, No. (%)	SE (95% CI)	*P* value^a^
HS vs sham	20 (37.7) vs 7 (13.2)	0.08 (0.11 to 0.38)	.004
MO vs sham	14 (26.4) vs 7 (13.2)	0.08 (0.01 to 0.26)	.09
HS vs MO	20 (37.7) vs 14 (26.4)	0.09 (−0.03 to 0.26)	.21
HS plus MO vs sham	34 (32.1) vs 7 (13.2)	0.07 (0.08 to 0.30)	.01

Abbreviations: HS, head-shaking; MO, mastoid oscillation.

^a^ Provided for χ^2^ test of independence significance at Bonferroni-corrected α = .0167.

In the per-protocol analysis, 20 of 47 patients (42.6%) in the head-shaking group, 14 of 49 patients (28.6%) in the mastoid oscillation group, and 7 of 46 (15.2%) in the control group showed the resolution of vertigo and nystagmus the following day. Comparison of therapeutic efficacy between the head-shaking group and sham group was statistically significant (*P* = .004; α = .0167).

In logistic regression analysis, the head-shaking group showed significantly higher odds of treatment success compared with the mastoid oscillation and control groups (OR, 2.50; 95% CI, 1.19-5.25; *P* = .02); compared with the head-shaking group, the mastoid oscillation group did not (OR, 1.06; 95% CI, 0.49-2.27; *P* = .88). Neither age (OR, 1.01; 95% CI, 0.98-1.05; *P* = .70) nor sex (OR, 1.14; 95% CI, 0.51-2.43; *P* = .73) was associated with treatment success. For the secondary end point, the immediate efficacy of 2 trials of each maneuver within 30 minutes, no significant difference was detected between treatments and sham groups (6 of 53 [11.3%] vs 4 of 53 [7.5%] vs 2 of 53 [3.8%]; *P* = .34; α = .05).

All the maneuvers were readily performed in all patients without serious adverse events. Overall, transition into another type of BPPV was observed in only 3 patients (1.9%; 1 in the head-shaking group and 2 in the sham group), and all showed a transition into PC-BPPV-ca.

## Discussion

The efficacy of treatments for PC-BPPV-cu has not yet been validated. Two previous studies have examined various maneuvers in PC-BPPV-cu.^18,19^ The Semont maneuver, the Epley maneuver, and the hybrid maneuver (modified Semont maneuver) were ineffective for treating 10 patients with PC-BPPV-cu after 1 week.^19^ In a previous randomized clinical trial including some authors of the present study,^18^ neither the Epley maneuver nor the Brandt and Daroff exercise succeeded in achieving a competent resolution rate. These results suggest that the treatment of PC-BPPV-cu would be more challenging than expected.

This randomized multicenter clinical trial compared the therapeutic efficacy of the head shaking and mastoid oscillation with a sham maneuver in patients with PC-BPPV-cu. Our study showed that the head-shaking maneuver is an effective treatment for PC-BPPV-cu, with a short-term success rate of 37.7% after 2 applications. However, the success rate of the head-shaking maneuver was lower for PC-BPPV-cu compared with HC-BPPV-cu (77%).^11^ The discrepancy in the success rates could be attributed to the different orientation of the ampulla between HC and PC when performing the horizontal head-shaking maneuver (Figure 1A). The HC ampulla is nearly perpendicular to the ground,^20^ and thus, the otolith of the HC cupula receives a significant amount of inertia during the horizontal head-shaking maneuver, which is transferred equally to both the utricular and canal sides of the cupula. However, the PC ampulla tilts posteriorly,^21,22,23^ resulting in less inertia being transferred to the PC cupula, particularly to the canal side. The hypothesis could explain the lower immediate efficacy of the head-shaking maneuver and the minimal conversion rate to canalolithiasis in our study. Additional repetitions of the head-shaking maneuver and techniques that thoroughly deliver inertia to the PC cupula may improve the resolution rate of PC-BPPV-cu, which requires validation in the future. Given the anatomical position of the PC ampulla and the constant rotational axis around the neck joint during the head-shaking maneuver, shaking the head and body en bloc with the head bent down by 45° would be a more appropriate technique. The potential benefit of the head-shaking maneuver may be its effectiveness in removing the otoliths from the cupula irrespective of the affected side and easy application in patients who have an unclear lesion side. However, the head-shaking maneuver would not be tolerable in some patients with cervical stiffness due to limited head mobility.

This study showed no effectiveness of the mastoid oscillation maneuver in treating PC-BPPV-cu. Mastoid oscillation has been applied in the HC-BPPV-cu with the hypothesis that it may facilitate the detachment of otoliths from the cupula.^12,24^ However, previous research on the efficacy of mastoid oscillation as a treatment option for HC-BPPV-cu^25^ has yielded conflicting results depending on various head position. Similar to our study, the mastoid oscillation in the seated position did not provide any additional benefit during BD exercise in patients with HC-BPPV-cu, who did not respond to both head-shaking and modified Semont maneuvers.^25^ In contrast, the mastoid oscillation performed while lying down and tilting the head by 25° to position the lateral semicircular canal vertically, or mastoid oscillation while turning the head by 135° and 90° toward the healthy side, were effective treatments for HC-BPPV-cu since they can detach otolith debris from both the canal and utricular sides of the cupula.^12,24^ In this regard, the mastoid oscillation with the head flexed downward by 135° and tilted backward by 45° after turning to the lesion side would be more plausible maneuvers for treating PC-BPPV-cu.

There are other underlying reasons that may explain the differing outcomes observed between the head-shaking and mastoid oscillation maneuvers. These may be attributable to different biophysical mechanisms or variations between the 2 methods. The therapeutic efficacy of head-shaking in treating BPPV-cu may be attributed to its alternating acceleration and deceleration power, which has been demonstrated to detach the debris from the cupula.^25^ In contrast, the effect of mastoid oscillation may be analogous to the effect that shaking a bottle of ketchup has on the flow of the ketchup (ie, a shaking effect).^12,26^ Mastoid oscillation itself may also minimize the adherence of the otolith debris and decrease its angle of repose relative to the sidewalls of the semicircular canal.^12,27^ To dislodge the otolith debris from the cupula, it would be more efficacious to use dynamic alternating acceleration and deceleration rather than static shaking of the cupula.

### Limitations

This study has some potential limitations. First, since this study only observed immediate and short-term resolution due to the high number of losses to follow-up at 1 week (17 of 62 [27.4%]) in the previous study,^18^ we could not assess the long-term therapeutic efficacy of the head-shaking and mastoid oscillation maneuvers or the natural course of PC-BPPV-cu. A total of 10.7% of patients were lost to follow-up the day after treatment. It is imperative that future research endeavors focus on ascertaining the long-term treatment effects. This will facilitate the reduction of the incidence of follow-up loss through in-hospital treatment, in conjunction with the provision of economic reward. Second, the sham group also showed a resolution rate of 3.8% after a single application and 9.4% in the following day. Since there was little immediate resolution in the Epley maneuver and BD exercise group in the previous investigation,^18^ the initial step of the sham maneuver toward the healthy side may generate a force that detaches the otoliths from the cupula. Third, study was conducted solely within a Korean population, which limits the generalizability of the findings to other populations. Last, since all of our patients did not undergo imaging, some with central pathology may have been included in our study. However, central lesions are very rare in pure positional vertigo or dizziness, and patients with central positional vertigo mostly presented with positional apogeotropic horizontal or downbeat nystagmus.^28^

## Conclusions

This randomized clinical trial found short-term therapeutic efficacy of the head-shaking maneuver in treating PC-BPPV-cu. The head-shaking maneuver can be considered as a primary option for treating PC-BPPV-cu.

## Notes

Supplement 1.
Trial Protocol

Supplement 2.
Data Sharing Statement
