# Supplementary material for: Treatment Maneuvers in Cupulolithiasis of the Posterior Canal Benign Paroxysmal Positional Vertigo: A Randomized Clinical Trial
Source: JAMA Netw Open. 2025 Mar 19;8(3):e250972. doi: 10.1001/jamanetworkopen.2025.0972 (PMC11923721; doi:10.1001/jamanetworkopen.2025.0972)
Supplement: Supplement 1. — Trial Protocol [file jamanetwopen-e250972-s001.pdf]

Head shaking and Mastoid Oscillation Maneuvers in Posterior Canal-Benign Paroxysmal Positional Vertigo-Cupulolithiasis: A Randomized Clinical trial

Version No.: Ver 1.2

▪ STUDY SUMMARY

Title	Head Shaking and Mastoid Oscillation Maneuvers in Posterior Canal-Benign Paroxysmal Positional Vertigo-Cupulolithiasis (PC-BPPV-cu)
Supervision institution & Principal investigator	Department of Neurology, Pusan National University Hospital Kwang-Dong Choi (Professor)
Funding agency	Memorial Foundation for Dr. Suh Succjo by named Hyangseal, Korean Neurological Association (KNA-21-HS-08)
Study Purpose	The purpose of this study is to determine immediate and short-term therapeutic efficacies of head-shaking and mastoid oscillation maneuvers in PC-BPPV-cu
Study design	Multi-center, double blind, randomized controlled trial
Study period	IRB approval date ~ 4 years
Target population	Subjects with PC-BPPV-cu confirmed at the participating clinics during the study period.
Number of subjects	159 subjects (53 for each arm)
Vulnerable person	Not applicable
Investigational product	Not applicable
Usage and dosage	Not applicable

Study methods	1) Subjects with PC-BPPV-cu confirmed at each clinic will be randomly assigned (1:1:1) to the head shaking, mastoid oscillation, or control group using an interactive web response system.<br>2) A treatment maneuver will be performed once by a physician according to assigned treatments or sham group.<br>3) The treatment response will be determined by participating neurologists in each clinic without knowing the maneuver applied to each patient at 30 minutes after initial maneuver.<br>4) When the subjects will show positioning nystagmus, they will receive the previously applied maneuver again.<br>5) The subjects will undergo re-examination 30 minutes later and will be scheduled for follow-up the next day without additional treatment if they will still present with positional vertigo and nystagmus.
Target disease	Posterior Canal-Benign Paroxysmal Positional Vertigo-Cupulolithiasis
Inclusion/exclusion criteria	Patients who meet the following inclusion and exclusion criteria.<br><br><u>Inclusion criteria</u><br>1) A history of recurrent attacks of positional vertigo<br>2) Positional nystagmus beating torsionally with the upper pole of the eye to the lower ear and vertically upward (to the forehead) and lasting > 1 min which was evoked by Dix-Hallpike or half Dix-Hallpike maneuver<br>3) Absence of identifiable CNS disorders that could explain the positional vertigo and nystagmus.<br><br><u>Exclusion criteria</u><br>1) Younger than 20 years<br>2) Subjects with multicanal BPPV, cervical spinal problems, or cognitive dysfunction<br>3) Subjects for whom, according to the investigators' opinions,

	participating in this clinical trial would pose a serious danger to their well-being or would result in unreliable outcomes at the end of the study.<br>4) Subjects with severe, acute, chronic, progressive, or degenerative diseases that may affect this clinical trial.<br>5) Subjects who do not agree to participate in this clinical trials
Efficacy evaluation	The resolution of both positional vertigo and nystagmus
Safety evaluation	Not applicable
Visit schedule	Subjects will be scheduled to return to the clinic the next day if the vertigo and nystagmus will not resolve after two randomized maneuvers on the day of the visit.
Statistical analysis	Any difference in the treatment response would be determined using ANOVA and Chi-square test between the treatments and control groups.
Expected effects	The head shaking and mastoid oscillation maneuvers would be recommended as initial treatment options for PC-BPPV-cu.

▪ STUDY PROCEDURES

Study procedure	V1		V2 (V1+1day)	
	Treatment group	Control group	Treatment group	Control group
			When the subjects still have positional vertigo and nystagmus	
Informed consent	X	X		
Demographics assessment	X	X		
Inclusion/exclusion criteria	X	X		
Medical history	X	X		
Assessment of dizziness history	X	X		
Neuro-otological examinations	X	X		
Positional test	X	X	X	X
Canalith repositioning maneuver	X	X		
Determination of treatment response	X	X	X	X
End of study			X	X

▪ STUDY PROTOCOL APPROVAL FORM

1. TITLE

Head Shaking and Mastoid Oscillation Maneuvers in Posterior Canal-Benign Paroxysmal Positional Vertigo-Cupulolithiasis: A Randomized Controlled Trial

2. STUDY SITE

SITE 1: Pusan National University Hospital

SITE 2: Pusan National University Yangsan hospital

SITE 3: Keimyoung University Dongsan Hospital

SITE 4: Ulsan University Hospital

SITE 5: Dong-A University Hospital

SITE 6: Dae-dong Hospital

3. SPONSOR

Pusan National University Hospital

4. FUNDER

Memorial Foundation for Dr. Suh Succjo by named Hyangseal, Korean Neurological Association (KNA-21-HS-08)

5. BACKGROUND AND RATIONALE

1) STUDY BACKGROUND

Benign paroxysmal positional vertigo (BPPV) is a disease characterized by short and repetitive rotational dizziness caused by head or posture transformation, and is one of the most common causes of dizziness. [1-5] It is caused by the otoconia present in the utricle escaping into the semicircular canal due to degenerative changes in the inner ear or other causes. Migraine headaches, head injuries, other internal ear diseases, vestibular neuropathy, Meniere's disease, and stroke that invades cerebrovascular vessels such as the anterior inferior cerebellar artery (AICA) can also be important causes of secondary BPPV. Although the mechanism of separation from the utricle of the otoconia is unclear, it has been also reported to be related to autoimmune diseases, diabetes, and osteoporosis including accompanying inner ear disease and thyroid disease. [4] The average incidence age is 50, and the incidence rate tends to increase as the age increases. It is known that the lifetime prevalence of BPPV is 2.4%, and the one-year incidence rate is about 0.6%.

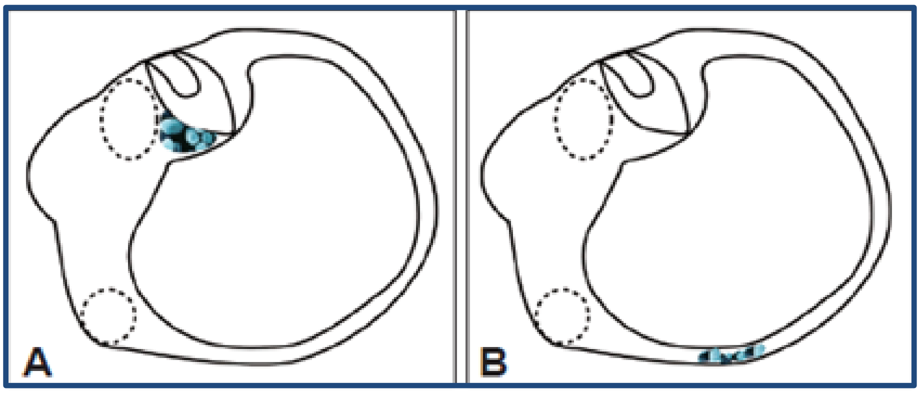

Figure 1. Mechanism of benign paroxysmal positional vertigo (BPPV). The detached otolith debris may be attached to the cupula (A: Cupulolithiasis) or free-floating in the semicircular canals (B: Canalolithiasis).

The condition in which otoconia are adhered to the cupula is referred to as cupulolithiasis, while the condition in which they are suspended within the semicircular canal is known as canalolithiasis (Figure 1).

Cupulolithiasis of the posterior canal benign paroxysmal positional vertigo (PC-BPPV-cu) produces a positional nystagmus, with the upper pole of the eye beating torsionally towards the lower ear and vertically upwards, which lasts longer (> 1 min.) than experienced in canalolithiasis of PC-BPPV (PC-BPPV-ca) during Dix-Hallpike or half Dix-Hallpike maneuver (Figure 2). This diagnostic criterion helps distinguish PC-BPPV-cu from other forms of BPPV.

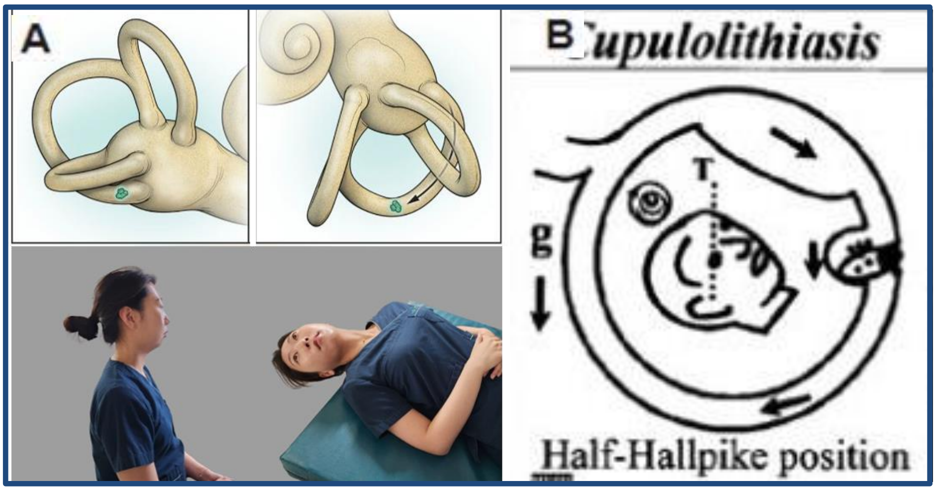

Figure 2. Diagnostic maneuvers of cupulithiasis type of posterior semicircular canal BPPV (PC-BPPV-cu). A. Dix-Hallpike maneuver B. Half Dix-Hallpike maneuver

BPPV was traditionally considered as a benign condition that often resolved spontaneously over time without specific treatment. On average, PC-BPPV resolved about 39 days later. However, for the effective management of BPPV and to alleviate dizziness, accurate diagnosis and canalith repositioning maneuvers (CPM) are recommended.

Previous randomized controlled trials have proven that head-shaking and mastoid oscillation maneuvers effectively treat cupulolithiasis of the horizontal canal benign paroxysmal positional vertigo (HC-BPPV-cu). However, no data exist regarding the therapeutic efficacy of these maneuvers in PC-BPPV-cu.

2) STUDY PURPOSE

The purpose of this study is to determine immediate and short-term therapeutic efficacies of head-shaking and mastoid oscillation maneuvers in PC-BPPV-cu.

6. PARTICIPANT ELIGIBILITY

1) DISEASE

: Posterior canal-Benign paroxysmal positional vertigo-cupulolithiasis (PC-BPPV-cu)

2) INCLUSION CRITERIA

A. A history of recurrent attacks of positional vertigo

B. Positional nystagmus beating torsionally with the upper pole of the eye to the lower ear and vertically upward (to the forehead) and lasting > 1 min which was evoked by Dix-Hallpike or half Dix-Hallpike maneuver

C. Absence of identifiable CNS disorders that could explain the positional vertigo and nystagmus.

3) EXCLUSION CRITERIA

A. Younger than 20 years

B. Subjects with multicanal BPPV, cervical spinal problems, or cognitive dysfunction

C. Subjects for whom, according to the investigators' opinions, participating in this clinical trial would pose a serious danger to their well-being or would result in unreliable outcomes at the end of the study.
D. Subjects with severe, acute, chronic, progressive, or degenerative diseases that may affect this clinical trial.
E. Subjects who do not agree to participate in this clinical trials.

4) PARTICIPANT RECRUITMENT PLAN

A. All patients who meet the inclusion and exclusion criteria for this study among patients diagnosed with BPPV after visiting the study site during the study period will be included.
B. The principal investigator of this study will not exclude patients who are likely to participate in this study based solely on race or socioeconomic status. Every effort will be made to ensure that as many patients as possible can participate in this study if the inclusion criteria are met, and the purpose of the study will be communicated to patients so that they can represent all patients with BPPV treated at the study site.

7. METHODS

1) STUDY METHODS

A. Subjects with PC-BPPV-cu confirmed and treated at each clinic will be randomly assigned to the treatment groups or to the control (sham) group.

B. A treatment maneuver was performed by a physician according to the assigned treatments or sham group.

C. Treatment response was determined by the participating neurologists at each clinic without knowing the maneuver applied to each patient 30 minutes after the initial maneuver.

D. If the patient had positional vertigo and nystagmus, the previously applied maneuver was repeated.

E. The patients underwent re-examination 30 minutes later and were scheduled for follow-up the next day without additional treatment if they still presented with positional vertigo and nystagmus.

2) TREATMENT MANEUVERS

The diagnosis and treatment (Canalith repositioning procedure) of participants who meet the inclusion/exclusion criteria are conducted from the baseline day (the first day) onwards. The choice of treatment is randomly assigned. Once the treatment method is selected, it is administered at each center accordingly.

A. Head shaking maneuver: In a seated position with the head slightly tilted forward by approximately 30 degrees, shake the head from side to side at a frequency of 3Hz for 15 seconds

B. Mastoid oscillation maneuver: Using a hand-held body massager (ID113, china), apply vibrational stimulation to the mastoid at a frequency of 100Hz. Each session of vibrational stimulation lasts for 1 minute, with a 1-minute rest period in between, and this cycle is repeated for a total of 3 times.

C. Sham maneuver: Lie quickly in the opposite direction of the lesion and then rise again after 1minute.

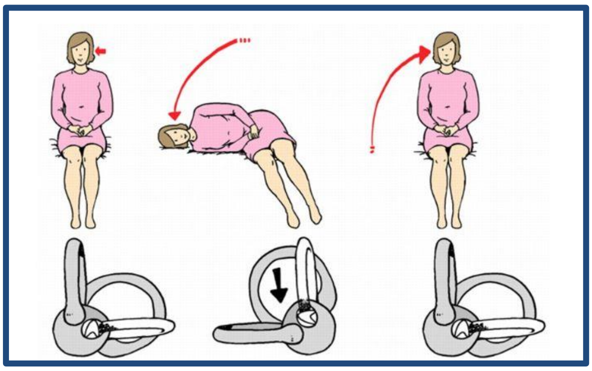

Figure 3. Sham maneuver

3) RANDOMIZATION, BLINDING, AND CODE-BREAKING

A. Each study site will be assigned a unique four-digit center code when enrolled. Participants considered for entry to the study at their screening visit will be identified by a unique within-center serially assigned participant number. Participant numbers will be assigned automatically by the trial management system and no attempt will be made to allocate numbers outside the system.

B. At their randomization visit (visit 0), participants who fulfill all the inclusion criteria and violate none of the exclusion criteria will be assigned a unique randomization number that will allow subsequent identification of their randomized treatment group allocation.

C. The randomization will be done using a concealed, computer-generated list of management assignments based on the predetermined simple randomization schedule provided by REDCap© (ver. 9.5.2, Vanderbilt University, USA).

D. The random allocation was designed in permuted blocks with a block size of 3.

4) ASSESSMENTS

A. The absence of both vertigo and nystagmus was required to determine a resolution.

B. Evaluation of treatment effect: The efficacy of treatment will be judged by an independent investigator blinded to the subject's information. If there is no improvement after the initial treatment, the patient will receive the same treatment once more. If there is still no improvement, the patient will visit the hospital again the following day for further evaluation.

5) TRIAL ENDPOINTS

A. Primary endpoint: The overall short-term resolution rate of positional vertigo and nystagmus in the following day.

B. Secondary endpoint: The immediate efficacy of two trials of each maneuver within 30 minutes.

6) BENEFITS AND RISK FOR THE PARTICIPANTS

A. By participating in this study, it will be possible to save time and medical expenses by immediate treatments. Even when symptoms persist, subjects will be allowed to visit the hospital the next day and receive treatment, which is a faster process than using the general treatment system. Therefore, there are no anticipated risks from participation in this study.

B. Subjects who visit the hospital due to persistent symptoms will receive a video-oculography test. The study team will be responsible for the medical fees of video-oculography.

7) DISCONTINUATION / WITHDRAWAL OF PARTICIPANTS

A. When subjects request discontinuation of the study

B. In other cases where the study must be stopped at the discretion of the researcher

8. STATISTICS

1) DESCRIPTION OF STATICS METHODS

The demographic distribution of study participants by treatment maneuvers were compared using the Chi-squared test and one-way analyses of variance (ANOVA). If the results of the Chi-squared test were significant, additional post-hoc tests were performed using Bonferroni adjusted alpha level of 0.0167 per test (0.05/3). Statistical analyses were performed using SPSS (version 27.0; SPSS, Chicago, IL, USA).

The primary outcome was analyzed using both intention to-treat and per-protocol methods. Patients who did not complete the assessment in the following day were excluded from the per-protocol analysis.

2) THE NUMBER OF PARTICIPNATS

The following are assumed to calculate the sample size.

A. Level of significance, α=0.0167
B. The ratio of the treatment and control group = 1:1:1
C. Power of the test, 1-β = 80%
D. In a previous study, we estimated that the proportions of patients with immediate resolution in PC-BPPV would 70% in the treatment groups, and 38% in the sham group.
E. The formula for calculating the number of samples and the calculation results are as follows.
    i. $n1=n2=[Z_{\alpha/2}\ \sqrt{2\bar{P}(1-\bar{P})} + Z_{\beta}\sqrt{P_1(1-P_1)+P_2(1-P_2)}]^2 / (P_1-P_2)^2$
    ii. n1= Sample size in the control group
    iii. n2= Sample size in the treatment group

iv. p1, p2= Expected outcome rates for each group

G. Based on these estimations, a sample size of 159 participants is obtained with a dropout rate of 5%.

H. The calculated sample size considers the drop rate, and in actual clinical research, more or fewer participants may be required than the calculated number of study subjects. When 30%, 60%, or 90% of the expected study subjects are achieved, a Data and Safety Monitoring Board meeting will be held to determine whether the number of participants is appropriate.

3) THE LEVEL OF STATISTICAL SIGNIFICANCE

$P < 0.05$

4) PROCEDURE FOR ACCOUNTING FOR MISSING, UNUSED, AND SUPURIOUS DATA

Missing values in parameters will be imputed with the Last Observation Carried forward.

5) INCLUSION IN ANALYSIS

The primary outcome would be analyzed using both intention-to-treat and per-protocol methods. Patients who can not successfully use the web-based system will be excluded from the per-protocol analysis.

6) PROCEDURE FOR REPORTING ANY DEVIATIONS FROM THE ORIGINAL STATISTICAL PLAN

This process will be conducted with a guidance of an IDMC responsible for monitoring safety and effect during the trial. The final study protocol and informed consent will be reviewed and approved by the corresponding health authorities and ethics

boards/institutional review boards for all participating study sites.

7) DATA SAFETY MONITORING PLAN (DSMP)

A. Responsible person

   i. Monitoring manager: Kwang-Dong Choi (PNUH)

   ii. Person in charge of monitoring: Eun Hye Oh (PNUYH)

B. Items

   i. study accruals: essential documents, ICF (informed consent form), CRF (case report form)

   ii. safety: encryption of data, appropriateness of data management, contrast source documents and study documents

C. Methods and interval

   i. The independent data monitoring committee (IDMC) regular review is once a year.

   ii. When 30% of the expected study subjects are recruited, an IDMC meeting will be held.

   iii. Every time a serious adverse event occurs, or when the monitoring manager deems it necessary, an irregular review will be held.

D. Reporting of adverse events, non-compliance, and reporting of unexpected problems

   i. Adverse events, unexpected problems, and non-compliance with the protocol found according to the monitoring results will be reported to the IRB within 15 working days after becoming aware of the details.

E. Criteria for study discontinuation

   : This study will be stopped under the following conditions

i. When the target number of study subjects has been reached.

ii. Cases where unexpected serious adverse events discovered during the study are continuously reported.

9. THE SAFETY PROTECTION OF SUBJECTS

1) RESEARCH ETHICS

A. All researchers comply with the research ethics and ICP-GCP specified in the Helsinki Declaration (revision in 2013) when conducting research.

B. This study will be conducted after IRB approval.

2) CONSENT PROCESS

A. The researcher who explains the study to subjects and obtains consent will be the principal investigator or a doctor authorized to obtain consent from the principal investigator. Delegation of obtaining consent is possible only to a researcher participating in this study.

B. The researcher must explain the study using language that is easy for the general public to understand and provide opportunities to subjects to ask questions and receive answers about the study.

C. In this process, there will be no possibility of coercion or undue influence, and it should be made clear that there will be no disadvantage to the subject even if he or she does not participate in this study.

D. This study does not include children and patients with cognitive impairment as the target group, so the consent form must be prepared by the subject himself/herself.

E. All subjects who have agreed to participate in this study will be provided with an explanation of the study, a copy of the consent form, and contact information for the researcher in charge.

3) INSURANCE

If the patient is harmed while taking part in this clinical trial as a result of negligence on the part of a member of the trial team, this liability coverage would apply. Non-negligent harm is not covered by the NHIC indemnity scheme. The PNUH, therefore, cannot agree in advance to pay compensation in these circumstances. In exceptional circumstances an ex-gratia payment may be offered.

4) PARTICIPANT CONFIDENTIALITY

A. The trial staff will ensure that the participants' anonymity is maintained, and even when the study results are published, the participants' identities will be kept confidential.

B. The personal information of the participants collected for this study will be the date of birth and sex.

C. All documents related to the study, such as the CRF, should be recorded and classified by the subject identification code and participants' initials rather than the participants' names.

D. Electronic data will be saved in a password-protected file, and documents that need to be kept as a paper will be stored in a locked documents box.

E. All documents related to the study will be kept for 3 years from the time the study is completed, and the data for which the storage records have been completed will be destroyed in accordance with Article 16 of the Enforcement Decree of the Personal Information Protection Act.

10. REFERENCE

1. Baloh RW, Jacobson K, Honrubia V. Horizontal semicircular canal variant of benign positional vertigo. Neurol. 1993;43:2542-2549.
2. Lempert T. Horizontal benign positional vertigo. Neurol. 1994;44:2213-2214.
3. Fife TD, Iverson DJ, Lempert T, et al. Practice parameter: therapies for benign paroxysmal positional vertigo (an evidence-based review): report of the Quality Standards Subcommittee of the American Academy of Neurology. Neurol. 2008;70:2067-2074.
4. Han BI, Oh HJ, Kim JS. Nystagmus while recumbent in horizontal canal benign paroxysmal positional vertigo. Neurol. 2006;66:706-710.
5. Von Brevern M, Radtke A, Lezius F, et al. Epidemiology of benign paroxysmal positional vertigo: a population based study. J Neurol Neurosurg Psychiatry. 2007; 78: 710-715.
6. Steenerson RL, Cronin GW, Marbach PM. Effectiveness of treatment techniques in 923 cases of benign paroxysmal positional vertigo. Laryngoscope. 2005;115:226-231.
7. Moon SY, Kim JS, Kim BK, et al. Clinical characteristics of benign paroxysmal positional vertigo in Korea: a multicenter study. J Korean Med Sci. 2006;21:539-543.

8. Oh SY, Kim JS, Jeong SH, et al. Treatment of apogeotropic benign positional vertigo: comparison of therapeutic head-shaking and modified Semont maneuver. J Neurol 2009;256:1330-1336.
9. Oh SY, Kim JS, Choi KD, et al. Switch to Semont maneuver is no better than repetition of Epley maneuver in treating refractory BPPV. J Neurol. 2017;264(9):1892-1898.
10. Lee SH, Kim JS. Benign Paroxysmal Positional Vertigo. J Clin Neurol. 2010;6:51-63
11. Kim JS, David SZ. Clinical practice. Benign paroxysmal positional vertigo. N Engl J Med. 2014;370(12):1138-47
12. Epley JM. Human experience with canalith repositioning maneuvers. Ann N Y Acad Sci. 2001;942:179-91
13. Kim JS, Oh SY, Lee SH, et al. Randomized clinical trial for apogeotropic horizontal canal benign paroxysmal positional vertigo. Neurol. 2012;78:159-166
14. Kim CH, Hong SM. Is the modified cupulolith repositioning maneuver effective for treatment of persistent geotropic direction-changing positional nystagmus? Otol. 2018;275:1731-1736.
15. Gold DR, Morris L, Kheradmand, et al. Repositioning Maneuvers for Benign Paroxysmal Positional Vertigo. Curr Treat Options Neurol. 2014;16:307.
