# Supplementary material for: Treatment Maneuvers in Cupulolithiasis of the Posterior Canal Benign Paroxysmal Positional Vertigo: A Randomized Clinical Trial
Source: JAMA Netw Open. 2025 Mar 19;8(3):e250972. doi: 10.1001/jamanetworkopen.2025.0972 (PMC11923721; doi:10.1001/jamanetworkopen.2025.0972)
Supplement: Supplement 2. — Data Sharing Statement [file jamanetwopen-e250972-s002.pdf]

Data Sharing Statement

Oh. Treatment Maneuvers in Cupulolithiasis of the Posterior Canal Benign Paroxysmal Positional Vertigo. *JAMA Netw Open*. Published March 19, 2025.
doi:10.1001/jamanetworkopen.2025.0972

Data
Additional Information: Clinical trial registration KCT0004756 https://cris.nih.go.kr
Data available: Yes
Data types: Deidentified participant data
How to access data: Data supporting the findings of this study are available from the corresponding author upon reasonable request. To access the data, please send an email to ist1335@hanmail.net.
When available: With publication

Supporting Documents
Document types: None

Additional Information
Who can access the data: anyone requesting the date
Types of analyses: for any purpose
Mechanisms of data availability: with investigator support
